# Artificial Intelligence Applications for Automated Data Extraction and Secondary Use of Clinical Information in Uro-oncology: A Systematic Review

**DOI:** 10.1016/j.euros.2026.02.006

**Published:** 2026-02-26

**Authors:** Julian Greß, Gordon Otto, Sebastian Sommer, Markus K. Schuler, Shahbaz Khan, Florian Schröder, Christoph Seidel

**Affiliations:** a Technical University of Munich, Munich, Germany; b MPiriQ Science Technologies GmbH, Munich, Germany; c MVZ Elisenhof, Munich, Germany; d Onkologischer Schwerpunkt am Oskar-Helene Heim, Berlin, Germany; e Department of Oncology, Hematology and Stem Cell Transplantation with Division of Pneumology University Medical Center Hamburg-Eppendorf, Hamburg, Germany

**Keywords:** Artificial intelligence, Natural language processing, Large language models, Clinical information extraction, Uro-oncology, Real-world data, External validation, Implementation science, Machine learning, Electronic health records

## Abstract

**Background and objective:**

Manual data extraction is a major bottleneck in uro-oncology, limiting research and quality assurance. Although artificial intelligence (AI) offers a scalable solution, the quality and generalizability of current evaluations remain unclear. This review aims to assess the performance, validation strategies, and real-world implementation of AI for automated data extraction in uro-oncology, encompassing a methodological spectrum from rule-based natural language processing to large language models, and to provide recommendations for rigorous evaluation standards.

**Methods:**

A systematic search of PubMed, Web of Science, and Embase was conducted through May 2025 following the Preferred Reporting Items for Systematic Reviews and Meta-analyses guidelines. The search was restricted to studies published from 2020 onward to focus on modern AI capabilities. Two reviewers independently screened records, extracted data, and assessed risk of bias using the Prediction model Risk Of Bias Assessment Tool (PROBAST).

**Key findings and limitations:**

Fourteen studies, encompassing between 100 and 66 532 patient records and approximately 120 000 individual clinical documents across genitourinary cancers, were included. AI models demonstrated high technical performance on structured data extraction, with reported F1 scores frequently exceeding 0.90. However, 86% (12/14) relied solely on internal validation; only two studies reported external validation. Nine studies (64%) described workflow benefits such as improved efficiency and reduced manual abstraction time. Most studies were retrospective and single center, with heterogeneous reporting that precluded a meta-analysis. Evidence for clinical application, cost effectiveness, calibration, and long-term sustainability was limited. These limitations highlight the need for robust external validation, human-in-the-loop verification, improved calibration reporting, equity assessments, and an implementation-science approach.

**Conclusions and clinical implications:**

AI shows strong potential for automating data extraction in uro-oncology, but clinical translation is limited by insufficient external validation and methodological heterogeneity. A shift from isolated performance metrics toward demonstrated robustness and trustworthy clinical application is needed to support reliable clinical use.

**Patient summary:**

In this study, we reviewed how artificial intelligence (AI) is being used to extract information automatically from medical reports on urological cancers. We found that most AI systems can identify important clinical details very accurately, but these are usually tested in only one hospital and not yet shown to work reliably in other settings. This means that while AI has great potential to save time and improve data quality, more testing in everyday clinical practice is needed before it can be used safely and routinely.

## Take Home Message

Artificial intelligence systems can extract clinical information from uro-oncology documents with consistently high technical performance, frequently achieving F1 scores above 0.90 and demonstrating meaningful efficiency gains. However, this systematic review reveals that clinical readiness remains limited: (1) 85% of models lack any external validation, (2) calibration and interpretability are rarely reported, and (3) studies are predominantly single center, retrospective, and methodologically heterogeneous. This disconnect between accuracy and trustworthiness underscores the need for a decisive shift toward rigorous external and temporal validation, human-in-the-loop verification, transparent calibration reporting, fairness assessments, and implementation-science frameworks. Only through such standards can artificial intelligence–driven data extraction become reliable, safe, and deployable in routine uro-oncological care.

## 1 Introduction

Clinical data in urological oncology are increasingly abundant but often locked within unstructured text, such as pathology and radiology reports. Manual abstraction of key variables—including Gleason score; tumor, node, metastasis (TNM) stage; and margin status—is a well-documented bottleneck for research, quality assurance, and clinical trial matching [1], [2]. Artificial intelligence (AI), particularly natural language processing (NLP), offers a scalable solution to automate this information extraction, enabling the secondary use of real-world data [3], [4], [5], [6], [7], [8], [9], [10]. In this review, AI is conceptualized as a technological hierarchy, spanning deterministic rule-based algorithms to probabilistic machine learning (ML) and generative foundation models.

While the potential of AI has been reviewed broadly across various applications in uro-oncology—from imaging and pathology to treatment planning [11], [12], [13]—a significant evidence gap persists regarding the clinical implementation of specific tools. Particularly for the automated extraction of information from clinical text, critical questions concerning workflow integration, generalizability across different institutions, and real-world application remain largely unanswered [14], [15], [16]. Although the advent of large language models (LLMs) has accelerated development, it also introduced new challenges related to reliability, governance, and robustness [17], [18], [19]. Consequently, there is a pressing need to synthesize evidence that moves beyond mere performance metrics such as the area under the curve—toward evaluating real-world readiness and clinical application [20], [21].

To address this gap, we conducted a systematic review of AI applications for clinical information extraction from textual data across prostate, bladder, kidney, and testicular cancers. This review aims to: (1) map the landscape of AI methodologies applied, from traditional ML to modern LLMs; (2) catalog the specific data elements and document types targeted; and (3) critically appraise the evidence for implementation readiness, focusing on external validation, and reported workflow integration. By synthesizing this evidence, we provide a practice-oriented overview for clinicians, researchers, and developers on the current state and future directions of automated data extraction in urological oncology.

## 2 Methods

This systematic review was conducted and reported in accordance with the Preferred Reporting Items for Systematic Reviews and Meta-analyses (PRISMA) 2020 guidelines [22]. The protocol was prospectively registered in PROSPERO (ID: CRD420251116085).

Eligible studies had to meet the following criteria:

1. The population included patients with uro-oncological malignancies (prostate, bladder/urothelial, kidney, or testicular cancer).
2. The intervention consisted of an AI-based method (rule-based NLP, ML, deep learning, LLMs, or hybrid approaches) applied to extract information from clinical documents. For this review, AI encompasses rule-based NLP, classical ML, and LLMs. Notably, we observed that the included studies did not utilize a uniform definition of AI, representing a diverse range of methodological frameworks.
3. The outcomes included either technical performance metrics or implementation-related measures.
4. The study was an original peer-reviewed research article.

The exclusion criteria were studies with a non–uro-oncological focus, use of AI for purposes other than text extraction (eg, pure imaging radiomics), or nonoriginal research publications.

A comprehensive search of PubMed/MEDLINE, Web of Science Core Collection, and Embase was performed in May 2025 for publications from January 1, 2020 to May 31, 2025, adhering to the PRISMA 2020 guidelines. This search period was deliberately chosen to focus the analysis on the modern era of AI, characterized by rapid methodological advancements and the advent of LLMs relevant to current clinical implementation. The search strategy combined controlled vocabulary (eg, MeSH and Emtree) with free-text keywords relating to AI methods, clinical documentation, and uro-oncological malignancies (Table 1).

**Table 1:** Systematic search strategy and query components by database

Database	Date of search	Results	Search #	Search query
PubMed/MEDLINE	May 31, 2025	22	#1	(“Artificial Intelligence”[Mesh] OR “Machine Learning”[Mesh] OR “Natural Language Processing”[Mesh] OR “Deep Learning”[Mesh] OR “deep learning”[tiab] OR “machine learning”[tiab] OR “artificial intelligence”[tiab] OR “neural network*”[tiab] OR “natural language processing”[tiab] OR “large language model*”[tiab] OR “generative AI”[tiab] OR “transformer model*”[tiab])
			#2	(“Electronic Health Records”[Mesh] OR “Medical Records”[Mesh] OR “Health Information Systems”[Mesh] OR “electronic health record*”[tiab] OR “electronic medical record*”[tiab] OR “clinical documentation”[tiab] OR “patient record*”[tiab] OR “clinical note*”[tiab] OR “pathology report*”[tiab] OR “discharge summary*”[tiab])
			#3	(“Data Mining”[Mesh] OR “Information Storage and Retrieval”[Mesh] OR “data extraction”[tiab] OR “information extraction”[tiab] OR “text mining”[tiab] OR “real-world data”[tiab] OR “real world evidence”[tiab] OR “secondary use”[tiab] OR “data reuse”[tiab] OR “automated reporting”[tiab] OR “clinical data warehouse”[tiab])
			#4	(“Urologic Neoplasms”[Mesh] OR “Prostatic Neoplasms”[Mesh] OR “Urinary Bladder Neoplasms”[Mesh] OR “Kidney Neoplasms”[Mesh] OR “Testicular Neoplasms”[Mesh] OR “uro-oncology”[tiab] OR “urooncology”[tiab] OR “urologic oncology”[tiab] OR “prostate cancer”[tiab] OR “prostatic cancer”[tiab] OR “bladder cancer”[tiab] OR “kidney cancer”[tiab] OR “renal cancer”[tiab] OR “renal cell carcinoma”[tiab] OR “testicular cancer”[tiab])
			#5	#1 AND #2 AND #3 AND #4
Web of Science Core Collection	May 31, 2025	19	**–**	TS=(“Artificial Intelligence” OR “Machine Learning” OR “Natural Language Processing” OR “Deep Learning” OR “deep learning” OR “machine learning” OR “artificial intelligence” OR “neural network*” OR “natural language processing” OR “large language model*” OR “generative AI” OR “transformer model*”) AND TS=(“Electronic Health Records” OR “Medical Records” OR “Health Information Systems” OR “electronic health record*” OR “electronic medical record*” OR “clinical documentation” OR “patient record*” OR “clinical note*” OR “pathology report*” OR “discharge summary*”) AND TS=(“Data Mining” OR “Information Storage and Retrieval” OR “data extraction” OR “information extraction” OR “text mining” OR “real-world data” OR “real world evidence” OR “secondary use” OR “data reuse” OR “automated reporting” OR “clinical data warehouse”) AND TS=(“Urologic Neoplasms” OR “Prostatic Neoplasms” OR “Urinary Bladder Neoplasms” OR “Kidney Neoplasms” OR “Testicular Neoplasms” OR “uro-oncology” OR “urooncology” OR “urologic oncology” OR “prostate cancer” OR “prostatic cancer” OR “bladder cancer” OR “kidney cancer” OR “renal cancer” OR “renal cell carcinoma” OR “testicular cancer”)
Embase	May 31, 2025	29	#1	(exp artificial intelligence/ or exp machine learning/ or exp deep learning/ or “Artificial Intelligence”:ti,ab or “Machine Learning”:ti,ab or “Natural Language Processing”:ti,ab or “Deep Learning”:ti,ab or “neural network*”:ti,ab or “large language model*”:ti,ab or “generative AI”:ti,ab or “transformer model*”:ti,ab)
			#2	(exp electronic health record/ or “Electronic Health Records”:ti,ab or “Medical Records”:ti,ab or “Health Information Systems”:ti,ab or “electronic health record*”:ti,ab or “electronic medical record*”:ti,ab or “clinical documentation”:ti,ab or “patient record*”:ti,ab or “clinical note*”:ti,ab or “pathology report*”:ti,ab or “discharge summary*”:ti,ab or “operative note*”:ti,ab or “radiology report*”:ti,ab)
			#3	(exp data mining/ or “Data Mining”:ti,ab or “Information Storage and Retrieval”:ti,ab or “data extraction”:ti,ab or “information extraction”:ti,ab or “text mining”:ti,ab or “real-world data”:ti,ab or “real world evidence”:ti,ab or “secondary use”:ti,ab or “data reuse”:ti,ab or “automated reporting”:ti,ab or “clinical data warehouse”:ti,ab)
			#4	(exp urologic tumor/ or exp prostate cancer/ or exp bladder cancer/ or exp kidney cancer/ or exp testis cancer/ or “Urologic Neoplasms”:ti,ab or “Prostatic Neoplasms”:ti,ab or “Urinary Bladder Neoplasms”:ti,ab or “Kidney Neoplasms”:ti,ab or “Testicular Neoplasms”:ti,ab or “uro-oncology”:ti,ab or “urooncology”:ti,ab or “urologic oncology”:ti,ab or “prostate cancer”:ti,ab or “prostatic cancer”:ti,ab or “bladder cancer”:ti,ab or “urothelial carcinoma”:ti,ab or “kidney cancer”:ti,ab or “renal cancer”:ti,ab or “renal cell carcinoma”:ti,ab or “testicular cancer”:ti,ab)
			#5	#1 AND #2 AND #3 AND #4
			#6	#5 AND [article]/lim
Total records retrieved			70	

MeSH = Medical Subject Headings; tiab = title and/or abstract; TS = topic search.

This table provides a detailed and reproducible account of the search strategy employed to identify relevant literature. The search was conducted on May 31, 2025, across three major databases: PubMed/MEDLINE, Web of Science Core Collection, and Embase. The query architecture was built upon the logical combination (AND) of four distinct conceptual pillars: (1) terms for artificial intelligence and machine learning, (2) terms for clinical documents and health information systems, (3) terms for data extraction and secondary use, and (4) terms for uro-oncological cancers. The search leveraged a combination of database-specific controlled vocabularies (eg, MeSH in PubMed and Emtree in Embase) and a wide range of free-text keywords searched in titles and abstracts ([tiab], :ti,ab). The total number of records retrieved from all sources prior to deduplication was 70.

Two reviewers independently screened the titles and abstracts, followed by a full-text assessment of potentially relevant studies. Discrepancies in assessments were resolved through discussion or consultation with a third reviewer. Data were extracted independently by two reviewers using a structured form. Information collected included study design, cancer type, AI methodology, target variables, performance metrics, implementation outcomes, and validation approach. Disagreements during extraction were resolved through consensus. A narrative synthesis was planned.

The methodological quality of the included studies was assessed using tailored instruments. For prediction-oriented models, the Prediction model Risk Of Bias Assessment Tool (PROBAST) was applied. For information extraction pipelines, methodological quality was assessed based on reporting completeness, reproducibility, and validation rigor.

## 3 Results

### 3.1 Study characteristics

**Fig. 1:**
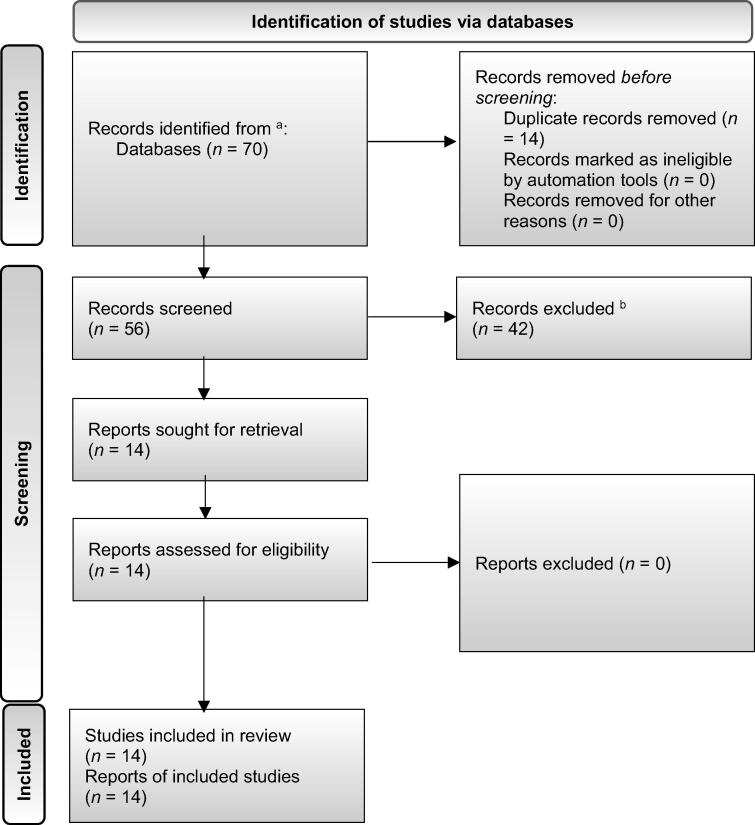
PRISMA 2020 flow diagram of the study selection process. The diagram illustrates the flow of information through the different phases of the systematic review. The process started with an initial search across three databases (PubMed, Web of Science, and Embase), which yielded 70 records. After removing 14 duplicates, 56 unique records were screened based on title and abstract, leading to the exclusion of 42 records. The full texts of the remaining 14 articles were assessed for eligibility, all of which met the inclusion criteria and were included in the final qualitative synthesis. The primary reasons for exclusion at the screening stage are detailed in the diagram. AI = artificial intelligence; PRISMA = Preferred Reporting Items for Systematic Reviews and Meta-analyses. ^a^ PubMed (*n* = 22), Web of Science (*n* = 19), and Embase (*n* = 29). ^b^ Wrong document type: nine studies, wrong AI application: 14, wrong data source/type: two, no AI method used: two, wrong focus: four, and other/undocumented: 11.

The initial search yielded 70 records. After duplicate removal, 56 unique records underwent title and abstract screening, which resulted in the exclusion of 42 records. The full texts of the remaining 14 articles were assessed for eligibility, and all were subsequently included in the final synthesis. The PRISMA flow diagram is shown in Figure 1.

The included studies originated primarily from the USA (*n* = 9, 64%), with the remainder from the EU (*n* = 3, 21%) and Asia (*n* = 2, 14%). Sample sizes ranged from 100 to 66 532 patient records (median 1794). Data sources were predominantly pathology reports (*n* = 7, 50%) and heterogeneous clinical documentation (*n* = 6, 43%). The most frequently studied malignancy was prostate cancer (*n* = 9, 64%). The detailed characteristics of each study are provided in Table 2.

**Table 2:** Synthesis of AI methodologies, technical performance, and validation status in uro-oncological data extraction

Author (year)	Country	Sample size	Cancer type	Document type	AI method	Key variables extracted	Performance metrics	Validation	Implementation outcomes
van Laar et al (2020) [34]	The Netherlands	100 patients	mRCC	Mixed EHR	Rule-based NLP	OS, PFS, ADEs	F1 >88%; almost identical OS/PFS curves	Internal (vs MR)	≈86% time reduction (86→12 min/patient)
Bozkurt et al (2022) [26]	USA	5461 patients	PC	Mixed EHR	Rule based and ML (SVM)	TNM stage	F1 (clinical T/N): 0.71–0.97	Internal (vs inter-rater kappa >0.85)	Imputed up to 71% of missing stage data
Guin et al (2022) [35]	USA	161 (final cohort)	PC (localized)	Mixed EHR	NLP (ConceptMapper)	ADT duration, T recovery	Accuracy (ADT duration estimation): up to 70%; data extraction F1: ≥0.95	Internal (vs manual curation)	Accuracy of the ADT duration estimate increased from 45% to 70%
Hsueh et al (2024) [30]	USA	1498 surgeries	RCC	Operative notes	LLM (GPT-4)	Laterality, surgery, approach, EBL, ischemia time	Accuracy (EBL): 90% (vs human 86%)	Internal (vs manual review)	GPT-4 as an efficient data extraction tool
Barman et al (2024) [33]	USA	9290 patients	Mixed	Mixed EHR	NLP (SciBERT based)/augmented curation (AC)	Immune-related AEs	F1: 0.844 (AI) vs 0.272 (structured diagnosis codes)	Internal (vs manual review)	More accurate AE detection with the AC model than standard codes
Park et al (2021) [36]	USA	750 reports	RCC, colon, lung	Pathology	TL (HCTC), ZSS	Tumor attributes	HCTC: F1 improvements of up to 0.1 micro-F1 0.04 macro-F1; ZSS: F1 improvement of up to 0.26 micro-F1 and 0.23 macro-F1	Internal (vs ground truth)	Halved required labeled data for similar performance
Hein et al (2025) [29]	USA	3520 reports (*n* = 2297 validated)	RCC, PC, breast	Pathology	LLMs (GPT-4o, etc.)	Histology, metastases	F1: 0.99 (histology), 0.97 (mRCC)	Both	Highly adaptable pipeline, reduces manual effort
Kirshner et al (2021) [25]	USA	66 532 patients	Mixed	Mixed EHR	ML (log. regression)	Metastatic status	Sens: 97%, Spec: 98% (after review)	External	>75% of cases classified automatically with high confidence
Huang et al (2023) [31]	Singapore	5772 reports	Uro-oncology	Pathology	Hybrid (rules + ML/DL)	11 clinical variables	Microaccuracy: 93%	Internal (vs registry)	Rule-based approach outperformed ML/DL
Fabacher et al (2020) [32]	France	982 (training)	PC	Pathology	ML (SVM) and RegEx	Incident case, Gleason, PSA	Precision: >99%, recall: ≈97%	Internal (vs registrar)	Halved registry processing time
Odisho et al (2020) [23]	USA	3232 reports	PC	Pathology	CNN/random forest	17 path. features	F1 (CNN): 0.97, Acc (RF): 0.93	Internal (vs dataset size)	High accuracy achievable with small datasets (∼128)
Yuan et al (2025) [27]	Taiwan	350 sim. cases	PC (stage IV)	Mixed imaging/path	LLMs	Risk assessment, TNM	Accuracy (GPT-4 RA): 90–92%	Internal (vs inter-rater kappa >0.78)	Potential as a clinical decision support screening tool
Altieri et al (2021) [24]	USA	500 reports	RCC, colon	Pathology	ML (SLA)	Tumor attributes	Micro-F1 improvement up to 0.10	Internal (vs baselines)	Increased sample efficiency (∼50% less data needed)
Miettinen et al (2021) [28]	Finland	389 (test set)	PC	Pathology	Rule based (RegEx)	Gleason score	F1: 0.95	Internal (vs manual processing)	768 person-hours (manual) vs 1–2 wk (dev.)

ADE = adverse drug event; ADT = androgen deprivation therapy; AE = adverse event; AI = artificial intelligence; CNN = convolutional neural network; dev. = development; DL = deep learning; EBL = estimated blood loss; EHR = electronic health record; HCTC = hierarchical convolutional text classification ; LLM = large language model; ML = machine learning; MR = manual review ; mRCC = metastatic renal cell carcinoma; NLP = natural language processing; OS = overall survival; PC = prostate cancer; PFS = progression-free survival; PSA = prostate-specific antigen; RA = risk assessment; RCC = renal cell carcinoma; RF = random forest; RegEx = regular expressions; Sens = sensitivity; sim. = simulated; SLA = supervised line attention; Spec = specificity; SVM = Support Vector Machine; T recovery = testosterone recovery; TL = transfer learning; TNM = tumor, node, metastasis; ZSS = zero-shot standardization.

Studies (*n* = 14) are categorized by clinical context, document source, and technical approach. The synthesis reveals a consistent trend of high technical performance (F1 scores frequently >0.90) across diverse AI methods, ranging from rule-based systems to LLMs. However, a systemic validation gap is evident: 86% (12/14) of studies relied exclusively on internal validation, while only two studies performed external validation. Quantifiable implementation outcomes (eg, workflow efficiency or data completeness) were reported in 64% (9/14) of the included studies.

### 3.2 Overview of key findings

**Fig. 2:**
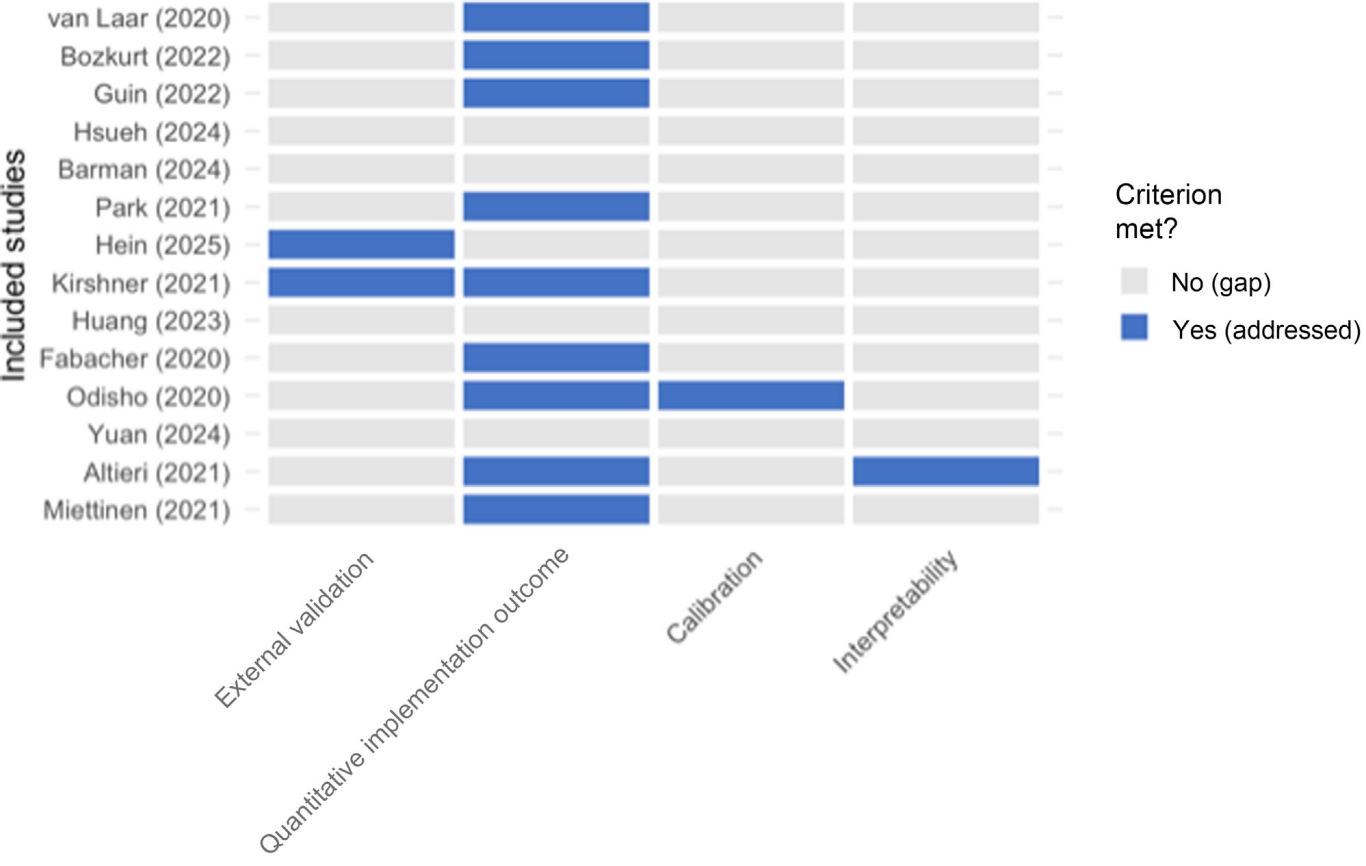
Visualizing the translational gap: assessment of clinical readiness beyond technical accuracy. This heatmap provides a visual synthesis of the review’s central finding by evaluating each of the 14 included studies against four critical dimensions of trustworthiness and real-world applicability: external validation, reporting of quantitative implementation outcomes, model calibration, and interpretability. Each row corresponds to an included study, and the columns represent the assessment criteria. Blue cells denote that a criterion was met, while gray cells signify a gap. The resulting pattern starkly reveals the translational gap: while a majority of studies (nine of 14 studies) reported on quantitative implementation outcomes, there are a profound deficit in external validation (addressed by only two of the 14 studies) and a near-complete absence of reporting on model calibration and interpretability—the key prerequisites for trustworthy clinical deployment.

Model performance across the 14 included studies was consistently high, with reported F1 scores frequently exceeding 0.90 and accuracy measures being above 90%. Most models—spanning rule-based systems, classical ML, and LLMs—achieved near-human or registry-level concordance for structured data fields such as TNM stage, histology, or Gleason score. Despite this encouraging technical performance, the validation landscape remained narrow: 12 of 14 studies (86%) relied exclusively on internal validation. Only two studies performed any form of external validation, confirming generalizability across institutions or data sources. Implementation outcomes were reported in nine studies (64%), most often quantifying workflow efficiency gains or improved data completeness. These findings reveal a clear disconnect: while the AI-based data extraction systems achieve high technical accuracy, their external validity and real-world robustness remain insufficiently established (Fig. 2 and Table 3).

**Table 3:** Quantified implementation benefits of AI-based data extraction in uro-oncology

Benefit category	Specific result	Quantitative impact	Study(ies)
Workflow efficiency	Reduced chart review time	86% reduction (from 86 to 12 min/patient)	[34]
	Accelerated cancer registry reporting	50% reduction in registration time	[32]
	Replacement for manual large-scale work	768 person-hours (manual) vs 1–2 wk (development)	[28]
	Reduced manual review need	>75% of cases automatically classified	[25]
Data completeness and quality	Imputation of missing staging data	21–71% of missing TNM data recovered	[26]
	Improved accuracy of clinical variables	ADT duration accuracy increased from 45% to 70%	[35]
	Enabling follow-up research	Dataset used for hypothesis validation	[35]
Resource optimization	Reduced annotation effort	High performance (F1 score >0.90) with only ∼128 reports	[23]
	Increased data efficiency	Comparable performance with ∼50% less labeled data	[24], [36]

ADT = androgen deprivation therapy; AI = artificial intelligence; TNM = tumor, node, metastasis.

This table synthesizes the reported implementation outcomes from the reviewed literature, focusing specifically on quantifiable benefits. It systematically categorizes these advantages into three primary domains: workflow efficiency, data completeness and quality, and resource optimization. The table highlights the tangible impact of AI tools beyond theoretical performance, showcasing significant reductions in manual labor (eg, an 86% reduction in chart review time), enhanced data integrity (eg, recovery of 21–71% of missing TNM data), and improved efficiency in model development (eg, achieving high performance with approximately 50% less annotated data). These findings underscore the practical value and return on investment for clinical and research applications.

### 3.3 Methodological quality and risk of bias

The methodological quality of the included studies was assessed formally, applying the PROBAST for prediction-oriented models. The overall risk of bias was judged to be high across most studies, with critical concerns concentrated in the validation and analysis domains. In the validation domain, a high risk of bias was assigned to 86% (12/14) of studies, primarily due to the exclusive use of internal validation methods. The absence of external validation was the most significant driver of bias, severely limiting the assessment of model generalizability; only two studies performed external validation. The risk of bias in the analysis domain was also high or unclear for most studies. The key methodological shortcomings included the failure to assess or report model calibration (13/14 studies) and the infrequent reporting of interannotator agreement (3/14 studies) to ensure a reliable ground truth. In the participants domain, the majority of studies were rated at a high risk of bias due to their single-center, retrospective design, which limits the applicability of the models to diverse patient populations.

Beyond the primary validation approach, the assessment of methodological quality also focused on model reliability and interpretability, and the integration of human oversight. A notable example was the work by Odisho et al [23], which placed a central focus on model reliability. They demonstrated that while their extraction models were initially calibrated poorly, application of isotonic calibration improved the trustworthiness of uncertainty estimates significantly, reducing the expected calibration error from values as high as 0.278 to as low as 0.033 or lower for all extraction fields. Methodological quality also encompassed model interpretability. Altieri et al [24] directly addressed this by designing their Supervised Line Attention model to be inherently interpretable. By first predicting the most relevant lines of text before making a final classification, their system allows end users to instantly verify the evidence for a given prediction. A crucial aspect of methodological quality was the integration of a human-in-the-loop (HITL) verification step. Kirshner et al [25] quantitatively demonstrated its value by showing that a final user review reduced the overall error rate of their classification tool from 4% to 2% in a real-world external validation.

The rigorous validation of the ground truth (interannotator agreement) was reported in only three studies (21%). Bozkurt et al [26] demonstrated high consistency between reviewers with Cohen’s kappa of 0.85 for clinical and 0.95 for pathological staging. Similarly, Yuan et al [27] reported high inter-rater reliability (kappa 0.78–0.90) between two expert urologists, with disagreements resolved by consensus. Miettinen et al [28] validated their manual extraction process on a subset of 200 texts, finding only negligible discrepancies (295 vs 288 scores identified) between independent processors. Conversely, Hein et al [29] explicitly excluded formal inter-reviewer metrics due to task complexity, while Hsueh et al [30] reported agreement metrics solely between the AI model and humans, leaving the variability within the human reference standard unquantified.

### 3.4 Methodological landscape and performance

The included studies employed a spectrum of AI methodologies, from rule-based systems to LLMs. Rule-based and hybrid systems proved effective for well-defined, structured tasks; Miettinen et al [28] achieved an F1 score of 0.95 for complex Gleason score expressions, and Huang et al [31] reported overall microaccuracy of 93%. Classical ML models (eg, Support Vector Machine [SVM] and logistic regression) were applied successfully to classification tasks such as automated cancer case identification [32] and metastasis classification [25]. More complex semantic challenges were addressed by deep learning models. Barman et al [33] used a SciBERT-based model to identify immune-related adverse events, achieving an F1 score of 0.844. LLMs demonstrated strong zero- or few-shot capabilities, with Hein et al [29] achieving a macroaveraged F1 score of 0.99 for histological subtypes, calculated on a primary validation subset of *n* = 2297 reports for which structured reference data were available.

Yuan et al [27] reported minimal performance differences for simple staging but significant disparities in complex risk assessment (where GPT-4-turbo exceeded 90% accuracy). Similarly, Hsueh et al [30] found high accuracy (91%) for extracting surgical approach but poor performance (31%) for ischemia time. This performance gap is likely attributable to the high narrative variability in operative reports; while terms such as “robotic” or “open” are standardized, ischemia time is often described through heterogeneous phrasing. For instance, GPT-4 struggled to recognize “off-clamp” as a null value and failed to aggregate cumulative clamping intervals—such as an initial 30 min followed by an additional 10 min—emphasizing a current deficiency in interpreting clinical context beyond simple string matching.

### 3.5 Clinical application and implementation efficiency

Regarding workflow efficiency, four studies documented substantial time savings. Fabacher et al [32] reduced the processing time for new cancer registrations by 50% by using an SVM to preidentify incident cases from a high volume of pathology reports. This allowed registrars to transition from primary manual screening to a verification-focused workflow, where biological data (eg, prostate-specific antigen and Gleason score) were prefilled via text mining. An 86% reduction in per-patient extraction time (from 86 to 12 min) was achieved by van Laar et al [34]. Miettinen et al [28] calculated that a manual task requiring 768 person-hours could be replaced by an automated pipeline developed in one to two person-weeks. Kirshner et al [25] demonstrated that their tool could automatically classify metastatic status for over 75% of cases with high confidence, allowing human experts to focus on the remaining complex cases.

Data completeness and quality improvements were also well documented. Bozkurt et al [26] imputed 21–32% of missing clinical stage data and, for patients with missing pathological stage, recovered 71% of missing T stages from the unstructured pathology reports. The validity of this extraction is supported by the model’s high performance on the test set (F1 score 0.94) against a ground truth established with high interannotator agreement (kappa 0.95). Guin et al [35] increased the accuracy of the estimation of androgen deprivation therapy duration from 45% (using structured data alone) to 70% by combining structured and unstructured data. The resulting enhanced dataset enabled a clinical outcome study consistent with the established literature.

Several studies demonstrated that AI can reduce the burden of data annotation. Odisho et al [23] found that model performance plateaued (weighted F1 score >0.90) with as few as 128 annotated reports. Similarly, Altieri et al [24] and Park et al [36] showed that their advanced methods achieved performance comparable with that of baseline models with approximately half the labeled data. These findings on sample efficiency indicate that sophisticated model design can lower the barrier to entry for clinical AI implementation.

## 4 Discussion

### 4.1 Principal findings

This systematic review exposes a central illusion in the current state of AI in uro-oncology: high technical performance (eg, F1 scores >0.90) equates to clinical application. Our analysis of 14 studies suggests that while technical performance is high, significant translational hurdles remain before these tools can be integrated routinely into clinical practice.

The primary obstacle identified is not feasibility, but a profound lack of trustworthiness. This review argues that trustworthiness is not a menu of options; it is an evidence chain built on robust external validation (missing in 86% of studies), proof of model calibration, and auditable interpretability. As summarized in Section 3.2, our findings show that this chain is broken at its first and most critical link, stalling progression and preventing safe real-world deployment.

### 4.2 The methodological spectrum: tradeoffs and implications

Our analysis confirms significant definitional heterogeneity: the included studies did not adhere to a shared taxonomy of AI, with approaches ranging from deterministic white-box rule–based NLP to latent-space deep learning and generative models. This lack of standardization complicates direct performance comparisons, as AI may refer to simple string matching in one context and probabilistic reasoning in another. The methodological spectrum identified in this review reveals a landscape of tradeoffs where transparency has often been sacrificed for performance without sufficient compensatory validation.

Rule-based systems [26], [28] represent a white-box ideal. While their transparency is a significant strength, their brittleness limits scalability across evolving clinical documentation and diverse reporting styles. In contrast, classical ML and deep learning models [33] offer superior flexibility and semantic understanding, enhancing data efficiency significantly [23]. However, this shift introduces a “black-box” dilemma, where model inscrutability necessitates a reliance on robust external validation—a step almost universally omitted in the current literature.

LLMs [27], [29], [30] represent a further shift toward prompt engineering. This flexibility, however, introduces the risk of nonauditable hallucinations. The substantial performance variability observed in our findings [30] serves as a reminder that LLM reliability remains highly task contingent. Ultimately, as models become less interpretable, the demand for rigorous external validation becomes the non-negotiable prerequisite for clinical trust.

### 4.3 Beyond accuracy: the critical gaps in validation, calibration, and interpretability

The most damning finding of this review is the systemic failure of validation. With 86% of studies relying solely on internal validation, the field’s claims of high performance are built on institutional sand, leaving generalizability as an unanswered, high-risk question. This call for rigor is not new [13], but our analysis demonstrates that years of methodological awareness have failed to translate into practice. The two studies [25], [29] that performed external validation are critical outliers, proving that generalizability is possible but remains shockingly rare.

However, this validation gap is compounded by two other critical deficits: calibration and integrity of the gold standard itself.

First, a model without calibration assessment on independent test sets is a clinical liability. While models are inherently calibrated to their training data, this does not guarantee accurate probability estimates on unseen data. A high F1 score is dangerously misleading if the associated confidence scores are unreliable. Furthermore, the reliance on F1 scores presents a challenge in interpretability, particularly for probabilistic ML models where the metric is highly sensitive to the classification threshold. The output of such models is typically a probability ranging between 0 and 1, which must be dichotomized into a binary outcome based on a specific cutoff. However, explicit reporting and justification of these probability thresholds were generally lacking across the included studies. Without standardized threshold definitions, a high F1 score in one study may not be directly equivalent to another, as it may reflect a different tradeoff between sensitivity and specificity rather than superior model performance.

The work by Odisho et al [23], which used isotonic calibration to fix this, should not be a standout example; it should be a mandatory step for any model claiming clinical readiness. Second, clinical trust is incompatible with black-box systems. The inherently interpretable model from Altieri et al [24], which shows users why a prediction was made, provides a necessary mechanism for end-user verification.

### 4.4 Quantifiable value: from efficiency gains to enabling new research

Despite the validation crisis, the potential value of these tools is not in dispute. The nine studies that quantified outcomes provide compelling, tangible evidence of what is at stake. Beyond oncology, similar AI-driven extraction frameworks have already demonstrated success in other medical domains, such as the automated identification of cardiovascular risk factors from primary care notes or the extraction of phenotypic data for rare disease registries, suggesting a broader feasibility for the methodologies discussed here.

The reported efficiency gains are not trivial. The 86% reduction in per-patient review time [34] or the autonomous classification of over 75% of cases [25] could alleviate the administrative burden of manual data entry, potentially allowing clinicians to focus more on patient-centered management. For instance, the semiautomated identification of critical parameters—such as surgical margins in prostate cancer or the presence of muscle-invasive disease in bladder cancer, or the extraction of warm ischemia time from partial nephrectomy notes—could assist in streamlining multidisciplinary tumor board preparations and the timely initiation of adjuvant treatment protocols. Finally, all validation is meaningless if the gold standard is flawed. The trustworthiness of any metric depends on the ground truth; yet, only three studies (21%) reported interannotator agreement. Ideally, the trustworthiness of any AI model depends on a reliable ground truth. Our review shows that high-quality manual annotation is feasible: Bozkurt et al [26] and Yuan et al [27] achieved high interannotator agreement (kappa >0.78) through rigorous definitions and expert consensus. However, this level of rigor remains the exception. In many studies, either the human baseline is assumed to be perfect without validation (eg, Hsueh et al [30] only measured AI-to-human agreement) or formal agreement metrics are omitted entirely due to the complexity of the extraction task, as noted by Hein et al [29]. This lack of ground-truth validation in the majority of studies represents a latent risk of bias where model performance might be under- or overestimated depending on the subjectivity of the single human reviewer.

More profoundly, NLP serves as an engine for generating new clinical evidence. While several studies completed datasets [26], the work by Guin et al [35] is a powerful example of this potential. They transformed an incomplete dataset into a research-grade resource and conducted a survival analysis consistent with the established literature. This demonstrates the transformative role of NLP: moving beyond simple data extraction to enabling the generation of real-world evidence that was previously infeasible.

### 4.5 Overcoming the data annotation bottleneck

A major practical barrier to developing clinical AI is the time and expertise required for data annotation. Encouragingly, several studies show that this barrier may be lower than assumed. Odisho et al [23] found that model performance began to saturate with as few as 128 annotated reports. Furthermore, advanced architectures [24], [36] demonstrated high performance with roughly half the labeled data of baseline models. These findings are critical; these signal that the barrier to entry for developing robust AI is not necessarily massive data, but smarter methodology.

### 4.6 Limitations

This review’s conclusions are constrained by the primary studies themselves, which we judge to be at a high risk of bias.

First, substantial methodological and clinical heterogeneity across the included studies precluded a quantitative synthesis or meta-analysis. This heterogeneity spanned AI methodologies, target variables, reference standards, performance metrics, and validation strategies, making statistical pooling inappropriate. This is not merely a statistical limitation but a finding in itself, reflecting the absence of standardized benchmarks and reporting practices in the field. Furthermore, this heterogeneity prevented a formal assessment of the certainty of evidence (eg, via the Grading of Recommendations, Assessment, Development, and Evaluations [GRADE] framework) for the reported outcomes. Furthermore, this heterogeneity prevented a formal assessment of the certainty of evidence (eg, via the GRADE framework) for the reported outcomes.

Second, by restricting our search to peer-reviewed, English-language publications, we are likely presenting an optimistic view. The true validation gap in unpublished or gray literature is presumably far worse. This restriction also reinforces a critical deficit: the complete absence of evidence for multilingual performance, limiting all findings to English-speaking health care systems. While English is the dominant language of scientific publication, in the specific context of AI, language itself is considered the data. Models trained on English syntax and medical terminology cannot be assumed to generalize to French or German clinical reports without specific retraining and validation. This focus reinforces a critical evidence gap regarding the performance of these tools in multilingual European settings, where heterogeneous documentation styles and a lack of standardized report structures pose additional challenges to successful external validation.

Third, the restriction of our search to the period from 2020 to 2025 was a deliberate choice to capture the era of modern AI architectures, such as transformers and LLMs. While this ensures relevance to the current technology, it excludes earlier foundational work in rule-based extraction. Finally, our May 2025 search cutoff means that we are in a constant race against innovation, particularly from LLMs. However, this focus on peer-reviewed literature is an intentional filter against preprint hype, grounding our conclusions in evidence that has survived formal scrutiny.

### 4.7 Ethical, regulatory, and governance considerations

Deployment is not only a technical problem, but one of clinical safety and governance too. Misclassification by AI tools presents direct hazards; for example, an incorrect extraction of pT3 margin status could lead to the omission of necessary salvage therapies, while errors in TNM stage extraction could inappropriately disqualify patients from intensive systemic regimens or clinical trials. Furthermore, the false identification of perioperative complications or the incomplete extraction of critical operative parameters could lead to an inaccurate assessment of surgical quality and patient outcomes. Regulatory frameworks such as the EU's AI Act and the U.S. Food and Drug Administration's software as a medical device guidance will classify many of these tools as high-risk medical devices, demanding traceability and postdeployment monitoring. Ethically, their outputs must be contestable. This requires auditable data provenance and non-negotiable HITL verification, not just for patient safety, but also as prerequisites for building clinician trust and ensuring alignment with data privacy mandates such as General Data Protection Regulation and the Health Insurance Portability and Accountability Act. The data extraction template used for this review is available from the corresponding author upon reasonable request.

### 4.8 Implications for clinical practice and future research

For near-term adoption, hybrid systems combining transparent rules with ML for complex tasks appear to be most practical. However, the true transformative potential may lie in redefining workflows entirely. Recent proof-of-concept studies explore a just-in-time clinical analysis paradigm, where LLMs bypass structured databases to perform complex analyses directly from raw text on demand [37].

To bridge the gap between today’s validation crisis and the trustworthy future, the field must pivot. Future research and development must be defined by the following non-negotiable priorities:

1. *Prioritize robust external and temporal validation*: Shift focus from single-institution internal validation to rigorous external validation across diverse health care systems. Models must be proved to be robust across different health care systems (geographic validation) and time periods (temporal validation).
2. *Mandate HITL verification*: Design all systems as semiautomatic pipelines. HITL is a non-negotiable safeguard for patient safety, error correction, handling ambiguity, and building clinical trust.
3. *Report model calibration and not just accuracy*: Move beyond F1 scores. Calibration must be a standard, reported metric for any model intended for clinical use. An uncalibrated confidence score is misinformation.
4. *Systematically audit for algorithmic equity*: Conduct fairness audits to assess model performance across demographic and linguistic subgroups. Models validated only on English-speaking, homogenous populations are not generalizable; these are inequitable.
5. *Focus on implementation science*: Measure and report on workflow integration, user acceptance, and economic impact. The true metric is time saved, cost reduced, and downstream data quality improved, not just the model’s accuracy.

## 5 Conclusions

AI demonstrates high-performing potential to automate information extraction in uro-oncology. Our review confirms that this potential is, as yet, unrealized. The field is currently stalled, celebrating technical performance while ignoring a systemic validation crisis. This chasm between proof of concept and proof of implementation is no longer acceptable. Bridging it requires a paradigm shift: from a focus on isolated metrics to a rigorous, holistic evaluation of real-world application, generalizability, and trustworthiness. The goal is no longer to build a model that works, but to prove that it is safe, auditable, and reliable. Only then can we transform data capture from a manual bottleneck into an automated byproduct of routine clinical care.

  ***Author contributions*:** Christoph Seidel had full access to all the data in the study and takes responsibility for the integrity of the data and the accuracy of the data analysis.

  *Study concept and design*: Seidel, Greß, Schuler, Schröder, Khan.

*Acquisition of data*: Greß, Otto, Seidel.

*Analysis and interpretation of data*: Greß, Seidel.

*Drafting of the manuscript*: Greß, Seidel, Khan.

*Critical revision of the manuscript for important intellectual content*: Otto, Schuler, Schröder, Khan, Sommer.

*Statistical analysis*: Seidel, Greß, Schröder, Khan.

*Obtaining funding*: None.

*Administrative, technical, or material support*: Schröder, Khan.

*Supervision*: Seidel.

*Other*: None.

  ***Financial disclosures:*** Christoph Seidel certifies that all conflicts of interest, including specific financial interests and relationships and affiliations relevant to the subject matter or materials discussed in the manuscript (eg, employment/affiliation, grants or funding, consultancies, honoraria, stock ownership or options, expert testimony, royalties, or patents filed, received, or pending), are the following: Markus K. Schuler, Shahbaz Khan, and Florian Schröder hold shares in MPIRIQ Science Technologies GmbH. Julian Greß, Gordon Otto, Christoph Seidel, and Sebastian Sommer receive honoraria from MPIRIQ Science Technologies GmbH.

  ***Funding/Support and role of the sponsor*:** None.
